# Essentials of the Principles and Practice of Medicine

**Published:** 1867-09

**Authors:** 


Essentials of the Principles and Practice of Medicine. A Handy-book for Students and Practitioners. By HENRY HARTSHORNE, M.D., Professor of Hygiene in the University of Pennsylvania, etc., etc., etc. Philadelphia: HENRY C. LEA. 1867.

This is a small-sized octavo volume, of 417 closely printed pages. It opens with an introductory chapter on Medical Systems or Theories, which gives a brief but interesting resume of medical history. Then follows four sections under the head of Principles of Medicine. The first is devoted to General Pathology; the second to Semeiology; the third to General Therapeutics; and the forth to Nosology. These sections occupy about 100 pages. Part Second is devoted to Special Pathology and Practice, and embraces a very brief consideration of all the diseases usually included in works on practical medicine. It closes with several pages of Formula referred to in the body of the work, and a copious index. As a sylabus or abstract of practical medicine and pathology, this little work of Dr. HARTSHORNE is the best that has come under our observation.

For sale by S. C. GRIGGS & Co. Price, $2.63.

